# A “modified Obel” method for the severity scoring of (endocrinopathic) equine laminitis

**DOI:** 10.7717/peerj.7084

**Published:** 2019-06-07

**Authors:** Alexandra Meier, Melody de Laat, Christopher Pollitt, Donald Walsh, James McGree, Dania B. Reiche, Marcella von Salis-Soglio, Luke Wells-Smith, Ulrich Mengeler, Daniel Mesa Salas, Susanne Droegemueller, Martin N. Sillence

**Affiliations:** 1 Earth, Environmental and Biological Sciences School, Queensland University of Technology, Brisbane, QLD, Australia; 2 Australian Equine Laminitis Research Unit, School of Veterinary Science, The University of Queensland, Gatton, QLD, Australia; 3 Animal Health Foundation, Pacific, MO, USA; 4 School of Mathematical Sciences, Queensland University of Technology, Brisbane, QLD, Australia; 5 Boehringer-Ingelheim Vetmedica, Ingelheim am Rhein, Germany; 6 Motion Equine Podiatry Consulting, Melbourne, Vic, Australia; 7 Veterinary Practice for Horses, Hamminkeln, Germany; 8 Veterinary Practice Datteln, Datteln, Germany; 9 Practice for Horses and Pets, Gehrden, Germany

**Keywords:** Laminitis, Lameness, Diagnostics, Horses, Equine metabolic syndrome, Insulin

## Abstract

**Background:**

Laminitis is a common equine disease characterized by foot pain, and is commonly diagnosed using a five-grade Obel system developed in 1948 using sepsis-related cases. However, endocrinopathic laminitis is now the most common form of the disease and clinical signs may be mild, or spread across two Obel grades. This paper describes a modified method which assigns scores to discreet clinical signs, providing a wider scale suitable for use in a research setting.

**Methods:**

The “modified Obel” method was developed using an iterative process. First, a prototype method was developed during the detailed observation of 37 ponies undergoing a laminitis induction experiment. The final method was refined and validated using video footage taken during the induction study and from a clinical trial of naturally occurring endocrinopathic laminitis cases. The Obel method was deconstructed and key laminitis signs were evaluated to develop a three-stage, five criteria method that employs a severity scale of 0–12. Veterinarians (*n* = 28) were recruited to watch and assess 15 video recordings of cases of varying severity, using the Obel and “modified Obel” methods. The inter-observer agreement (reproducibility) was determined using Kendall’s coefficient of concordance (Kendall *W*) and Krippendorf’s alpha reliability coefficient. A total of 14 veterinarians repeated the exercise 2–4 weeks after their original assessment, to determine intra-observer agreement (repeatability), assessed using a weighted kappa statistic (*kw*). Agreement between methods was calculated by converting all “modified Obel” scores to Obel grades and calculating the mean and distribution of the differences.

**Results:**

The “modified Obel” and Obel methods showed excellent and similar inter-observer agreement based on the Kendall *W* value (0.87, *P* < 0.001 vs. 0.85, *P* < 0.001) and Krippendorf’s alpha (95% CI) value (0.83 [0.53–0.90] vs. 0.77 [0.55–0.85]). Based on the *kw* value, the “modified Obel” method also had substantial repeatability, although slightly less than the Obel method, (0.80 vs. 0.91). Excellent agreement between the methods was found, with the mean difference (95% CI), comparing the Obel grade, with the “modified Obel” score converted to an Obel grade, being −0.12 (−0.19 to −0.06) grades. The Obel and converted “modified Obel” grades were identical 62% of the time (259/420) and a difference of one grade (higher or lower) occurred in 35% of cases (148/420).

**Conclusion:**

Both methods show excellent agreement, reproducibility and repeatability when used to diagnose endocrinopathic laminitis. The “modified Obel” method is a three-step examination process for severity-scoring of endocrinopathic laminitis, initially proposed for use within a research setting. When using the modified method a diagnosis of laminitis also requires clinical acumen. The allocation of scores for specific clinical signs should be particularly useful in research trials monitoring laminitis recovery.

## Introduction

Laminitis is a debilitating condition affecting all equids and is associated with several systemic diseases that may result in structural changes within the horse’s foot (Pollitt, 2016). It results in lameness, morbidity and mortality (Patterson-Kane, Karikoski & McGowan, 2018). Laminitis creates attrition within the horse industry with a recent study finding that 33% of all cases were humanely destroyed within 12 months of diagnosis (Luthersson et al., 2017).

Recently, endocrinopathic laminitis, associated with equine metabolic syndrome and pituitary *pars intermedia* dysfunction, has been acknowledged as the most prevalent form of the disease occurring in horses and ponies (Donaldson, Jorgensen & Beech, 2004; Karikoski et al., 2011; Patterson-Kane, Karikoski & McGowan, 2018). All forms of laminitis share the common clinical sign of foot pain, although it is recognized that the endocrinopathic form may have a milder clinical presentation, and be more insidious in onset than other forms of laminitis (McGowan, 2008; Pollitt, 2004). This is supported by histopathological evidence of laminitis occurring prior to the clinically apparent presentation of disease (Karikoski et al., 2015; Kawasako et al., 2009; Morgan et al., 2003).

The most widely accepted tool for diagnosing and categorizing the severity of laminitis is the Obel method, which was developed 70 years ago using cases of sepsis-related laminitis (Obel, 1948). The Obel method is favored in clinical research, having been used across a range of laminitis induction studies including the oligofructose method (Dern et al., 2017; van Eps & Pollitt, 2006), the prolonged euglycemic hyperinsulinemic clamp (Asplin et al., 2007; de Laat et al., 2010), and most recently, in a dietary model using non-structural carbohydrates (Meier et al., 2018a). Thus, the Obel method is applicable across several forms of laminitis with moderate repeatability and substantial reproducibility as demonstrated in one study of 25 ponies with acute pasture-associated laminitis (Menzies-Gow et al., 2010). Additionally, the Obel method has been compared to the clinical grading system (CGS) for evaluating equine lameness, and the visual analogue scale (VAS) which defines lameness severity along a 10 cm line according to a pain assessment from sound (clinically normal) to the worst possible pain (Viñuela-Fernández et al., 2011). Although these methods demonstrated high reliability, neither the CGS nor the VAS described the laminitis gait or allocated a laminitis score or grade. It’s also important to note, any laminitis grading system is assessing clinical foot pain, as opposed to the severity of the lamellar lesion. Laminitis pain has also been assessed using the Horse Grimace Scale which interprets facial-expressions, removing the need for locomotion; although promising results were seen in this study, the authors recommended further validation before clinical application (Dalla Costa et al., 2016).

Nevertheless, laminitis researchers are aware that diagnosing laminitis can be difficult due to the non-specific nature of clinical signs and lack of robust case definitions (Wylie et al., 2016). In particular, the difficulty in diagnosing mild cases of endocrinopathic laminitis was brought to light in our recent induction study (Meier et al., 2018a). A total of 37 ponies were fed a high non-structural carbohydrate diet over 18 days to induce laminitis. The insulin concentrations in response to this diet, measured over 4 h, were later used to determine an insulin risk threshold for the development of laminitis on this challenge diet. However, 10/11 ponies with insulin concentrations that exceeded the defined threshold for laminitis risk in the study displayed some clinical signs of laminitis such as a short, stilted gait, weight shifting and/or an increased digital pulse, but were not severe enough to meet the criteria for an Obel grade 1 diagnosis, as judged by the two laminitis experts (Meier et al., 2018a). Furthermore, this study found that cases of endocrinopathic laminitis did not always fit clearly within the individual Obel grades, with symptoms that often spread over two grades.

These findings highlighted that a modification of the Obel method would be useful to allow for scoring of individual clinical signs, with a graduated scale that could identify small changes over time. This could assist laminitis researchers to test treatment and preventive strategies, an important requirement as research progresses toward elucidating laminitis pathophysiology and developing efficacious therapies (Frank, 2018; Lane et al., 2017; Legere et al., 2018; Meier et al., 2018b).

Hence the aim of this study was to modify the Obel method of laminitis diagnosis into an examination that can characterize the severity of specific clinical signs, enabling the determination of a laminitis severity score. The modification needed to be suitable for use in monitoring the rate of recovery from laminitis in clinical trials assessing laminitis treatments.

## Materials and Methods

### Development of the “modified Obel” method

Ethics approval was granted from Queensland University of Technology (approval number 1600000891). The first step was to deconstruct the Obel method of laminitis diagnosis (Table 1) to determine the key diagnostic criteria. These criteria were then used to develop a prototype method for scoring laminitis using eight criteria on a 13-point scale (0–12), which could be translated back to the Obel system for comparative purposes (Table S1).

**Table 1:** The Obel method of laminitis diagnosis and severity grading (Obel, 1948).

Laminitis grade	Grade description
Normal	Horse appears sound
Obel grade 1	At rest, the horse shifts its weight between the forelimbs; the horse is sound at the walk, but the gait is stilted at the trot in a straight line and on turning
Obel grade 2	The gait is stilted at the walk and the horse turns with great difficulty, but one forelimb can be lifted
Obel grade 3	The horse is reluctant to walk and one forelimb can only be lifted with great difficulty
Obel grade 4	The horse will only move if forced to

The prototype method was used by laminitis experts Christopher Pollitt (CP) and Donald Walsh (DW) to diagnose and grade endocrinopathic laminitis severity via video recordings made during a laminitis induction study of 37 ponies (Meier et al., 2018a). The suitability of this prototype method was assessed post-study, including an analysis of the inter-observer agreement and the performance of individual laminitis indicators (criteria) in both laminitic and non-laminitic cases, to identify areas for improvement.

In addition, a comprehensive literature search was conducted to determine the most reliable and repeatable indicators of laminitis, and to exclude any poor performing indicators. Advice and feedback were also sought from laminitis experts regarding the clinical presentation of endocrinopathic laminitis and the usability of the prototype diagnostic method.

Based on this information, a final “modified Obel” method was developed (Table 2) and validated. To validate the modified method, 15 video recordings of horses and ponies ranging from normal (no laminitis) to severe endocrinopathic laminitis cases were compiled from recordings in either the laminitis induction study (*n* = 9) (Meier et al., 2018a), or as part of a subsequent clinical field trial using natural cases of endocrinopathic laminitis (*n* = 6). The recordings showed footage of weight shifting, forelimb lifting by an operator, walking, circling and trotting if the horse was physically able, with these variables examined in the order prescribed by the “modified Obel” method (Table 2). As digital pulses could not be examined by video recording, this variable was scored by the examining veterinarian on the day of laminitis diagnosis and video recording.

**Table 2:** The QUT method of laminitis diagnosis and severity scoring.

Order of examination	Criteria	Description	Points	Given points
Stage 1				
Examine horse standing	Weight shifting	No weight shifting	0	
		Weight shifting—including shifting weight between all feet;	2	
		lying down; and/or placing forelimbs in front of body		
Gently lift each foot up and put back down straight away	Forelimb lift	Prompt and willingly maintained (each forelimb)	0	
		Reluctant and maintained with difficulty (each forelimb)	1	
		Unable to lift foot/resists attempts to lift foot (each forelimb)	2	
Stage 2				
Conduct on hard surface. Walk horse approx. 30 m side-on to examiner	Gait at walk	Normal gait	0	
		Mild short, stilted gait—still moves willingly	1	
		Moderate short, stilted gait—reluctant/difficult to walk	2	
		Severe difficulty walking or unable to walk*	6	
Turn on a short lead clockwise and anti-clockwise	Gait at circle	Normal circling	0	
		Mild head rise, difficulty when turning, still moves willingly	1	
		Moderate, sharp head rise, reluctance/difficulty turning	2	
		Severe difficulty turning, slow and clearly painful	3	
Stage 3				
All feet must be square on ground (unless unable to stand)	Forelimb digital pulse	Normal—able to palpate, normal magnitude but not bounding	0	
		Increased magnitude or bounding digital pulse (each forelimb)	2	
			Total Score	

**Note:**

* Do not force horse to walk; skip gait at circle and continue with digital pulse.

The 15 recordings were then graded using both the Obel and the “modified Obel” methods by 28 experienced equine veterinarians who were recruited for the study. The veterinarians were blinded to any treatments or diagnosis made by others and were presented with the recordings in random order. A total of 14 respondents agreed to repeat the task after 2–4 weeks, when they were sent the recordings in a different order together with a new score sheet.

### Statistical analysis

Significance for all analyses was set at *P* < 0.05. Descriptive statistics were obtained using Prism 7.0 (GraphPad software, La Jolla, CA, USA). The grade and score data are presented as median (range).

Analysis of the inter-observer agreement (reproducibility) and intra-observer agreement (repeatability) was conducted using R 3.5.1 GUI 1.70 El Capitan build (R Foundation for Statistical Computing, Vienna, Austria). Two methods were used to assess the inter-observer agreement. Firstly, Kendall’s Coefficient of Concordance *W* (Kendall *W*) was determined for each diagnostic method using the DescTools R package (Signorell, 2018). This test provides an index of inter-observer agreement on a scale of 0–1, where 1 indicates maximum agreement (Robinson, 1957). The second method employed Krippendorff’s α reliability coefficient using the IRR R package (Garner, 2015), where an α score of 1 indicates perfect reproducibility, 0 indicates no reproducibility and <0 indicates positive disagreement between observers (Krippendorff, 1970). As no *P*-value was produced with this coefficient, a 95% bootstrap interval was performed to determine a 95% confidence interval for Krippendorff’s α.

Intra-observer agreement was estimated from a weighted Kappa statistic *kw* obtained using the IRR R package (Garner, 2015). The weighted kappa method assigns less weight to agreement as scores are further apart, and the scores are typically categorized as indicating a level of agreement that is: less than by chance (<0), slight (0.01–0.20), fair (0.21–0.40), moderate (0.41–0.60), substantial (0.61–0.80), or almost perfect (0.81–0.99) (Viera & Garrett, 2005). For the Obel method, the grade allocated was used to determine the weighted kappa statistic, but for the “modified Obel” method the criterion of digital pulse was removed from the overall score as repeatability of this was unable to be assessed via video. The intra-observer agreement for the “modified Obel” method was also assessed on an individual criterion basis to examine the total number of times (in percentage) that the same score was given in the repeated video assessment for weight shifting, forelimb lift, gait at the walk and gait at the circle.

To determine the level of agreement between the Obel and “modified Obel” methods, the “modified Obel” scores were converted to Obel grades in all cases (*n* = 420, 15 video recordings assessed by 28 veterinarians). For the purpose of conversion, “modified Obel” scores of 0–4, 5–6, 7–8, 9–10 and 11–12, were designated as Obel grades 0, 1, 2, 3 and 4, respectively. The conversion factors were determined in conjunction with laminitis experts according to the clinical signs that were common to the two methods, although precise matching was not possible (Tables 1 and 2). The degree of agreement was calculated by subtracting the Obel grade from the converted “modified Obel” grade, and the distribution of the difference was examined using a frequency histogram. The mean of the differences was calculated, as well as the 95% confidence interval using the standard deviation of the mean and the total sample size.

## Results

### Prototype method

Eight key diagnostic criteria used by the Obel method were identified, and these were used to develop a prototype scoring system (Table S1). When the prototype method was applied to the examination of 37 ponies, 14 were diagnosed with either Obel grade 1 or 2 laminitis. Of these animals, 14 had a bounding digital pulse; 12 gave a positive response to hoof-testing applied to the sole; 11 had a stilted gait at the walk; 10 shifted weight between the forelimbs, were lame at the trot and had difficulty turning in a circle; seven were unable to hold a 30 s foot lift on each forelimb; and 1 scored positive for reluctance to move.

Between the two experts (CP and DW) the percentage of inter-observer agreement for individual criteria in the prototype method was 100% for weight shifting and hoof testing; 93% for reluctance or willingness to move; 86% for foot lift; 79% for gait at the trot; 71% for gait at the walk and 64% for gait at the circle. For non-laminitic horses, the inter-observer agreement was between 96% and 100% for all criteria, highlighting that inter-observer agreement is higher in the examination of a horse without laminitis.

Overall, the prototype method performed well, however, of the 23 ponies used in the study that did not develop Obel grade 1 laminitis, 18 achieved a score of >0.5 but <3, which we categorized as “abnormal.”

### Refined “modified Obel” method

The refined “modified Obel” method is a simplified three-stage examination for laminitis, using five diagnostic criteria instead of the eight in the prototype method, and is shown in Table 2. The major modifications in this method include an expansion of the weight shifting criterion (beyond the classic alternating forelimb weight shifting), to include lying down and, while standing placing the forelimbs abnormally far in front of the body. The criterion of forelimb lift was shortened, so that an extended 30 s period of lifting was not required; simply the foot must be picked up and put back down straight away and scored accordingly (Table 2).

The assessment of the horse at the walk was expanded to encompass a mild, moderate or severe laminitis gait, while reluctance or willingness to move was incorporated to better describe the gait. In order not to force an animal with severe laminitis to move more than necessary, any case that scored a 6 at the walk (severe difficulty walking or unable to walk) was not subjected to circling. Similarly, trotting was removed as a criterion (Table 2).

A change in digital pulse magnitude was deemed an important criterion, and indeed in the induction study a bounding digital pulse was one of the earliest indicators of laminitis, occurring between 1 and 3 days before clinical laminitis was diagnosed (Meier et al., 2018a). It took an average of 4 days for a bounding pulse to disappear post-laminitis onset. Thus, a higher weight was given to the digital pulse criterion in the “modified Obel” method.

The procedure of hoof testing was removed because 18/37 (49%) clinically normal ponies had a positive response as graded by at least one expert (CP or DW) in the pre-study examination (before laminitis was incited) indicating an unacceptably high frequency of false positive scores (Meier et al., 2018a).

### Inter-observer agreement (reproducibility)

Both the Obel and “modified Obel” methods showed excellent inter-observer agreement between the 28 veterinarians. For Kendall *W*, the “modified Obel” and Obel methods gave similar results (0.87 and 0.85, respectively, both *P* < 0.001). Similarly, Krippendorff’s alpha reliability coefficient showed that both methods had good reproducibility with α = 0.83 (95% CI [0.53–0.90]) for the “modified Obel” method, and α = 0.77 (95% CI [0.55–0.85]) for the Obel method.

### Intra-observer agreement (repeatability)

A weighted kappa statistic was calculated for each of the 14 veterinarians who repeated the scoring exercise, as shown in Table 3. The average *kw* for the “modified Obel” method was 0.80, which represents substantial repeatability, and the average *kw* for the Obel method was slightly higher at 0.91. Using a simplified analysis, exact score matches for the four criteria (weight shifting, foot lift, walk and circle) in the “modified Obel” method occurred in 696/840 (83%) of scorings. For the individual criteria, weight shifting had the highest repeatability, with 192/210 (91%) of repeated scorings matching; foot lift was also very high with 180/210 (86%) of repeated scorings matching; gait at the walk was scored the same 170/210 (81%) of the time; and gait at the circle was scored the same 154/210 (73%) of the time. Veterinarians gave the exact same Obel grade to the same video in 155 of 210 scorings (74%).

**Table 3:** QUT scores and Obel grades (median, range) allocated to 15 videos of horses with varying laminitis severity (and normal animals) by 28 veterinarians.

	“modified Obel” score	Obel grade
Video 1	9 (6–11)	3 (2–3)
Video 2	6 (4–7)	1 (1–2)
Video 3	5 (2–6)	1 (0–2)
Video 4	8.5 (4–11)	2 (1–3)
Video 5	0 (0–2)	0 (0–1)
Video 6	7 (4–12)	2 (1–3)
Video 7	8 (3–10)	2 (1–3)
Video 8	0 (0–3)	0 (0–2)
Video 9	9 (7–12)	3 (2–4)
Video 10	7 (6–9)	2 (1–3)
Video 11	2 (1–4)	1 (0–2)
Video 12	11 (9–12)	4 (3–4)
Video 13	8 (5–11)	3 (2–4)
Video 14	7.5 (5–10)	2.5 (2–4)
Video 15	0 (0–3)	0 (0–1)

### Agreement between Obel and “modified Obel” methods

**Figure 1:**
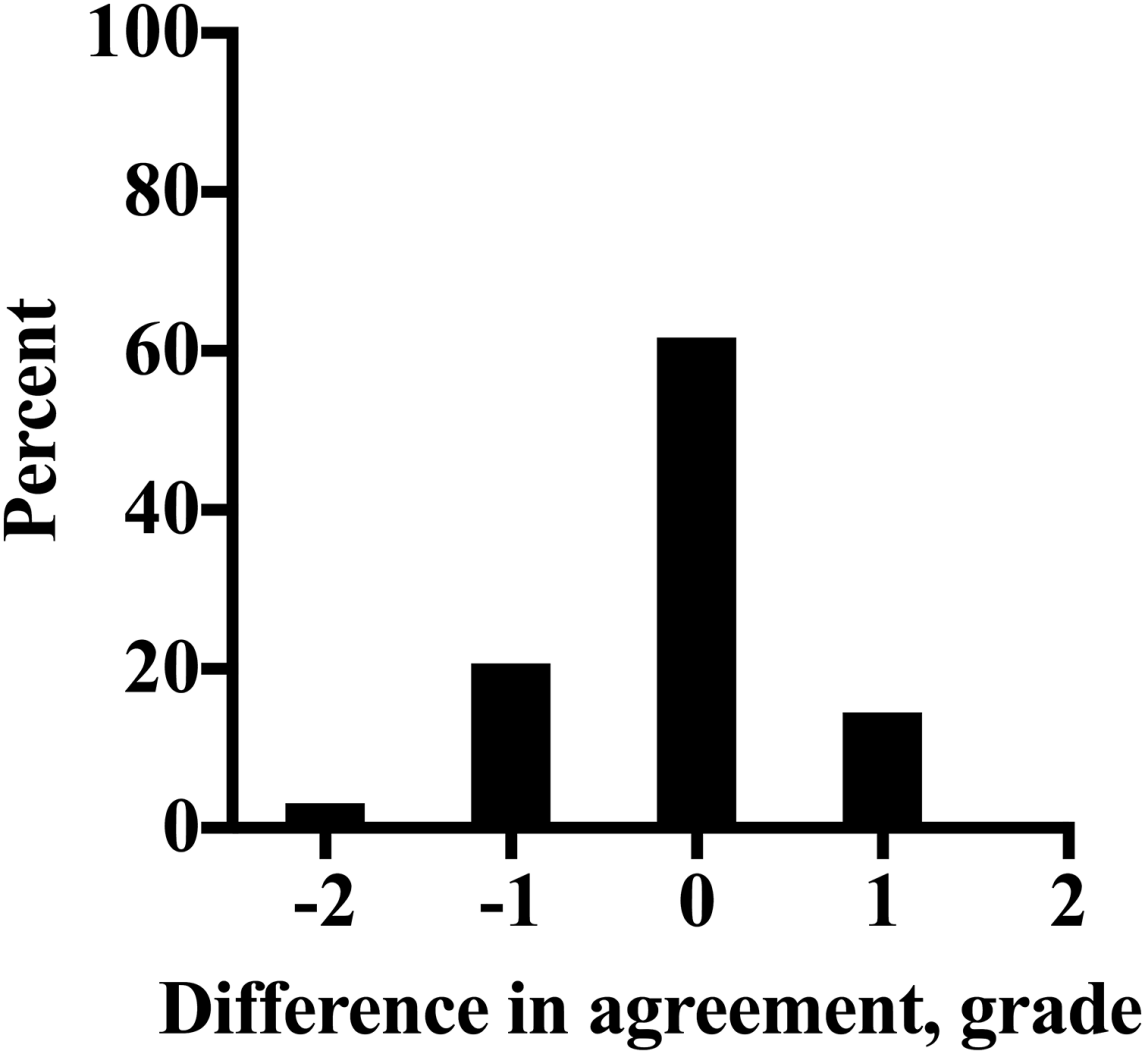
The distribution of difference in agreement between the Obel method of laminitis diagnosis and the QUT method converted to an Obel grade, as a percentage of all scorings (*n* = 420) determined by 28 veterinarians for 15 laminitis video recordings.

The refined “modified Obel” method and the Obel system were employed by 28 veterinarians to grade laminitis severity in 15 video recordings, with the results presented in Table 4. There was a clear agreement between the two methods when the “modified Obel” scores were converted to Obel grades. As shown in Fig. 1, the methods gave identical results in 62% (259/420) of scorings. In 35% (148/420) of scorings there was a difference of only one grade between the two methods, and a disparity of two grades found in only 3% of scorings (13/420). This result demonstrates a strong relationship between the two systems. Additionally, the mean difference (95% CI) between the 420 scorings showed that overall, the Obel method scored only −0.12 (−0.19 to −0.06) grades lower than the “modified Obel” method.

**Table 4:** Weighted kappa statistics (*kw*) for the QUT and Obel methods determining the intra-observer agreement (repeatability) for 14 veterinarians (raters) who graded a series of 15 laminitis videos twice.

	“modified Obel” method	*P*-value	Obel method	*P*-value
Rater 1	0.703	0.0059	1	0.0001
Rater 2	0.989	0.0001	1	0.0001
Rater 3	0.879	0.0005	0.812	0.0014
Rater 4	0.743	0.0011	0.86	0.0006
Rater 5	0.994	0.0001	1	0.0001
Rater 6	0.735	0.0042	0.905	0.0003
Rater 7	0.707	0.0053	0.931	0.0003
Rater 8	0.885	0.0005	0.907	0.0004
Rater 9	0.919	0.0004	0.814	0.0016
Rater 10	0.675	0.0083	0.911	0.0004
Rater 11	0.693	0.0068	0.842	0.0009
Rater 12	0.932	0.0003	0.905	0.0004
Rater 13	0.612	0.0171	0.925	0.0003
Rater 14	0.712	0.0054	0.894	0.0003
Average *kw*	0.80		0.91	

## Discussion

Overall, this study showed that both the Obel method and the new “modified Obel” method had excellent agreement, reproducibility and repeatability, when tested by 28 experienced equine veterinarians using 15 video recordings of endocrinopathic laminitis of varying severity (and normal animals). The Obel method was modified by using and analyzing a prototype method in a recent laminitis induction study, studying recent relevant literature and consulting expert opinion. The strength of the “modified Obel” method lies in its ability to score the severity of common clinical indicators of laminitis, which when combined, equate to an overall severity score (Table 2).

Importantly, in using the “modified” Obel method we do not prescribe a score or a level at which “clinical laminitis” is diagnosed. Although excellent agreement was found when the “modified Obel” method was converted to an Obel grade, the authors recommend using it as a stand-alone severity-scoring method, for example, in clinical trials monitoring laminitis recovery. A diagnosis of endocrinopathic laminitis needs to be made by a veterinary professional in conjunction with clinical acumen and supporting evidence such as history, as well as physical examination findings.

Critical to the development of this “modified Obel” method was the careful selection of the five-criteria included in the three-stage examination that would best capture all cases of endocrinopathic laminitis (Table 2). In examining the first criterion of weight shifting, our previous study found that the classic “alternating forelimb” weight shifting captured many, but not all of the laminitis cases (Meier et al., 2018a). Instead, other forms of weight shifting were seen as well, including lying down, and what is known as the “laminitis stance” where the forelimbs are placed in front of the body so as to bear more weight on the hindlimbs (Swanson, 1999), or to ease tension on the deep digital flexor tendons. Although this laminitis stance was seen in less than half of animals observed by Wylie et al. (2013), a further study found that animals that were not “shifting weight” between the forelimbs but had the “front feet placed in front of the body” were identified 94.2% of the time as laminitis cases (Wylie et al., 2016). Hence, expanding the description of weight shifting was necessary.

Lifting a forelimb by an operator was another contentious criterion, as it was argued on one side by some laminitis experts that the lift had to be difficult in each forelimb, as laminitis (with the exception of the supporting-limb form) is a bilateral disease. There is strong supporting evidence for this statement, as the laminitic gait at the walk is bilaterally stilted, and 92% of animals with this clinical sign did have laminitis (Wylie et al., 2016). Furthermore, in a previous study, laminitis was reported to occur in one forefoot in only 3.6% of cases (20/553), whereas it occurred in both forefeet 53.5% (296/553) of the time and in all four feet 41.4% (229/553) of the time (Wylie et al., 2013). Conversely, it was argued by other experts that clinical cases often have one forefoot that appears to be affected more severely than the other, and this may present as difficulty in lifting the contralateral limb, with the more affected limb appearing relatively easy to lift (in comparison). The “modified Obel” method requires that each forelimb must be easy to lift (forelimb lift prompt and willingly maintained in each forelimb) to score a 0 for this criterion. When conducting the examination, if the forelimb lift is not prompt and willingly maintained in either limb, a score of either 1 or 2 must be allocated to the animal for this criterion. Hence, even in cases where one forelimb is more painful than the other, the overall “modified Obel” score is not affected as a negative score (of 0) is only to be given if the foot lift is prompt and willingly maintained in each forelimb.

Difficulty turning in a circle is another common laminitis sign, occurring in more than 70% of laminitic horses (Wylie et al., 2013). The “modified Obel” method does examine gait at the circle, but it is not required to be assessed if the horse shows severe difficulty walking (Table 2). This is important for welfare reasons and to avoid forced locomotion exacerbating lamellar pathology. Limiting movement is a key treatment strategy preventing further damage to already weakened lamellar tissue (van Eps, 2010). Similarly, for the same reason, trotting is not examined, in line with veterinary practitioners avoiding trotting laminitic horses in over 50% of cases (Wylie et al., 2013).

A bounding digital pulse has been reported to be the most common clinical sign of laminitis (Wylie et al., 2013). Indeed, it was present in 91.2% of laminitis cases, as compared to 23.1% of non-laminitic lameness cases (Wylie et al., 2016). Forelimb digital pulse palpation is an easy task for an equine practitioner to perform, although one limitation of this study is that no data were obtained on the repeatability and reproducibility of this criterion.

Criteria that were included in the prototype method, but later excluded from the final method, were a reluctance to move and the response to hoof testing. The movement criterion was simply absorbed into the description of “gait at the walk” to better describe the laminitis gait. Hoof testing gave a positive response in too many ponies without clinical laminitis (48%) and so was removed (Meier et al., 2018a), despite the fact that the inter-observer agreement for this variable was 100%.

Further limitations of this study include the fact that bias could have occurred if the veterinarians were aware that one of the methods that they were using to assess laminitis cases was indeed the Obel method. Although no titles were given to the methods, and the cases were assessed using the methods in alternating sequence, many veterinarians are familiar with using the Obel method. In comparison to the Obel method, the “modified Obel” method is limited by not prescribing a threshold score at which clinical laminitis occurs. Whereas the Obel method has four well-defined grades, the “modified Obel” method scores individual criteria, and hence assigning a threshold at which laminitis occurs using the “modified Obel” method may result in mis-diagnosis (in the case of non-laminitis lameness cases) and has been avoided in this study. Furthermore, although all efforts were made to make the “modified Obel” method as objective as possible, there will still be a degree of subjectivity between examiners using this system, as witnessed in the intra-observer results where gait at the circle was given the same score 73% of the time, and gait at the walk 81% of the time, as opposed to weight shifting which had a 91% repeatability.

Other limitations include the number of cases explored, the number of veterinarians used to assess them, and the fact that video recordings were used instead of a live assessment for all cases. However, this is not the first study to utilize video analysis for laminitis diagnosis (Menzies-Gow et al., 2010; van Eps & Pollitt, 2009), and others have employed the method as an appropriate research tool for lameness diagnosis (Keegan et al., 1998; Fuller et al., 2006). Additionally, of the 15 videos utilized nine were filmed during a laminitis induction study using a high NSC diet, and hence may not replicate naturally occurring endocrinopathic laminitis entirely. However, it is the intention of the authors to assess the applicability of the “modified Obel” method within veterinary clinical practice using only natural cases in the future. Indeed, feedback was positive in terms of the “modified Obel” method applicability to clinical practice from veterinarians who participated in this study. In addition, the veterinarians who assisted this study by videoing clinical cases also utilized the “modified Obel” method on these cases, and reported that it was easy to understand and use.

The applicability of the “modified Obel” method in terms of owner use for very early detection of laminitis may also be limited, in that many of the signs upon which the criteria are based may not be present in the pre-clinical phase (with the exception of digital pulse). However, the method was not developed for this purpose, and owners and farriers need to be able to recognize symptoms such as pathologic changes in the hoof (dropped soles, abnormal growth rings on the external hoof wall and seedy toe) to detect pre-clinical laminitis (Floyd, 2007; Walsh, 2010).

## Conclusions

This study has shown that the “modified Obel” method has excellent repeatability and reproducibility when used to severity score endocrinopathic laminitis cases from both a laminitis induction study and naturally occurring cases. The “modified Obel” method is proposed for use in clinical laminitis trials, as a three-step examination that can allocate a severity score from 0 to 12 using individual clinical criteria. Future development of the “modified Obel” method will include its validation in tracking recovery from laminitis in clinical trials.

## Supplemental Information

10.7717/peerj.7084/supp-1 Supplemental Information 1 Prototype scoring system.

10.7717/peerj.7084/supp-2 Supplemental Information 2 Diagnostic scores for each variable.

## Additional Information and Declarations

### Data Availability

The following information was supplied regarding data availability: The raw data are available in a Supplemental File which contains the scores from each observer, for each diagnostic variable, in each animal.
