# Supplementary material for: A “modified Obel” method for the severity scoring of (endocrinopathic) equine laminitis
Source: PeerJ. 2019 Jun 7;7:e7084. doi: 10.7717/peerj.7084 (PMC6557244; doi:10.7717/peerj.7084)
Supplement: Supplemental Information 1 [file peerj-07-7084-s001.pdf]

**Supplementary table S1.** Prototype scoring system developed to diagnose laminitis in ponies in an endocrinopathic laminitis induction study (Meier et al., 2018a).

| Order of Assessment | Criteria | Description | Possible score |
|---|---|---|---|
| 1 | Weight shift | No weight shifting | 0 |
| | | Shifting weight between feet at rest | 1 |
| 2 | 30 s foot lift | Prompt and willingly maintained | 0 |
| | | Slow and maintained with struggle | 1 |
| | | Unable to maintain lift/resists attempts to lift | 2 |
| 3 | Gait at walk | Normal | 0 |
| | | Mild short stilted gait | 1 |
| | | Moderate or severe short stilted gait | 2 |
| 4 | Gait at trot | Normal | 0 |
| | | Pronounced short stilted trot | 1 |
| | | Unable to maintain a trot | 2 |
| 5 | Gait at circle | Normal | 0 |
| | | Head rises when circling towards lame foot | 1 |
| | | Head rises when circling towards both sides | 2 |
| 6 | Movement | Horse moves willingly | 0 |
| | | Horse reluctant to move | 1 |
| 7 | Digital pulse | Normal - difficult to palpate and not bounding | 0 |
| | | Abnormal - bounding digital pulse on palpation | 1 |
| 8 | Hoof testers | No response to solar pressure | 0 |
| | | Positive response to solar pressure | 1 |
